# Quantification of domestic cat hepadnavirus DNA in various body fluid specimens of cats: the potential viral shedding routes

**DOI:** 10.3389/fvets.2023.1248445

**Published:** 2023-09-04

**Authors:** Sabrina Wahyu Wardhani, Padet Tummaruk, Chutchai Piewbang, Somporn Techangamsuwan

**Affiliations:** ^1^The International Graduate Program of Veterinary Science and Technology, Faculty of Veterinary Science, Chulalongkorn University, Bangkok, Thailand; ^2^Animal Virome and Diagnostic Development Research Unit, Faculty of Veterinary Science, Chulalongkorn University, Bangkok, Thailand; ^3^Department of Obstetrics, Gynaecology, and Reproduction, Faculty of Veterinary Science, Chulalongkorn University, Bangkok, Thailand; ^4^Department of Pathology, Faculty of Veterinary Science, Chulalongkorn University, Bangkok, Thailand

**Keywords:** DCH, body fluid specimens, detection, horizontal transmission, shedding routes

## Abstract

Domestic cat hepadnavirus (DCH) belongs to the *Hepadnaviridae* family together with human hepatitis B virus (HBV) that remains to be a major health problem worldwide. The transmission of HBV infectious virion has been one of the essential factors that contribute to high number of HBV infection in humans. It has been long known that various body fluid specimens of human with chronic HBV infection contain HBV DNA and demonstrated to be infectious. In contrast to this knowledge, the detection of DCH in various body fluid specimens of cats, has not been reported. This study explored the detection of DCH DNA in various body fluid specimens of cats by quantitative polymerase chain reaction (qPCR) and investigated whether the detection of DCH DNA from broader routes was correlated with any genomic diversity by phylogenetic analysis. A total of 1,209 body fluid specimens were included, and DCH DNA was detected not only in 4.70% (25/532) of blood samples; but also in 12.5% (1/8), 1.14% (1/88), 2.54% (10/394), and 1.65% (3/182) of auricular swab (AS), nasal swab (NS), oral swab (OS), and rectal swab (RS) specimens, respectively. Furthermore, the level of DCH DNA detected in the blood was significantly correlated with DCH DNA detection in OS (*P* = 0.02) and RS (*P* = 0.04) specimens. Genomic analysis revealed that there was no notable genomic diversity within the complete genome sequences obtained in this study. In conclusion, this study highlighted the presence of DCH DNA in various body fluid specimens of cats, and the potential role of these specimens in DCH horizontal transmission within the cat population warrants further studies.

## Introduction

Human hepatitis B virus (HBV), a relaxed circular DNA virus, belongs to the *Hepadnaviridae* family and remains a major health problem worldwide as it is responsible for more than 800,000 deaths each year (1, 2). Despite the availability of an HBV vaccine, it is estimated that 360 million peoples suffer from chronic HBV infection with increased risk of life-threatening diseases such as cirrhosis and hepatocellular carcinoma (HCC) (3). In 2018, domestic cat hepadnavirus (DCH), an HBV relative, was detected in cats, and since then, several studies have reported the global detection of DCH in the blood and liver tissue of cats, with prevalence ranging from 0.78 to 18.5% (4–10). A concern regarding the possible role of DCH in the development of chronic liver disease in cats has been raised and investigated in several studies, and strikingly, an early study revealed that DCH was detected and localized only in the liver tissue of cats with chronic hepatitis and HCC, and no detection was made in the liver biopsy of healthy cats and cats with cholangitis and biliary carcinoma (5). Moreover, recent studies agreed that DCH was strongly associated with increased liver enzyme activities suggestive of hepatitis (7, 11), even after ruling out other possible viral infections that commonly cause hepatopathy in cats (9). The possible role of DCH in the development of hepatic disturbances and chronic hepatitis and HCC in cats, therefore, cannot be neglected.

The transmission of an infectious virion, specifically from chronically infected individuals that are asymptomatic serves as a challenge that contributes to the high HBV infection rate in humans. HBV is mainly transmitted horizontally by blood-related infection and sexual contact and vertically from the infected mother to her newborns (12). These days, however, there is growing evidence of HBV DNA detection from other sources, such as the saliva, sweat, tears, feces, and cerumen of infected individuals, with saliva and tears having been demonstrated to be infectious in human or animal models (12–15). In contrast to the growing knowledge about HBV transmission routes in humans, the detection of DCH in various body fluid specimens of cats has rarely been reported. Capozza et al. investigated the detection of DCH from broader routes, including oral, conjunctival, preputial, and rectal swabs from a cat, but no positive result was obtained, despite the prolonged DCH viremia status of the cat that remained for 11 months (16). With the increasing trend of multi-cat settings where close contact and fighting is inevitable, it is crucial to assess the potential transmission of DCH from various body fluid specimens other than blood. Therefore, the aim of this study was to explore the presence of DCH in the blood and other body fluid specimens, including auricular swab (AS), nasal swab (NS), oral swab (OS), rectal swab (RS), and urine obtained from cats from various provinces in Thailand and to determine whether the presence of DCH DNA in various body fluids is correlated with any genotypic difference using full-length genomic characterization.

## Materials and methods

### Sample collection

To investigate the presence of DCH in various clinical samples, we collected several body fluid specimens including blood, AS, NS, OS, RS, and urine from animal hospitals and cat shelters in Thailand. Clinical samples were collected from cats that underwent examination for a general health checkup or were appointed to receive chemotherapy or continuous treatment for chronic diseases. All specimens were collected with the prior owner's consent. The details of specimens collected from each cat are presented in Supplementary material S1.

Blood samples were collected in EDTA blood collection tubes, whereas AS, NS, OS, and RS specimens were collected using sterile cotton swabs and immersed in 0.6 mL of sterile 1× PBS in 1.5-mL Eppendorf tubes. Urine was collected using a non-invasive procedure by the free-capture method. All specimens were subsequently stored at −80°C prior to the extraction procedure.

All investigative procedures were conducted in accordance with the Animal Research: Reporting of In Vivo Experiments (ARRIVE) guidelines and regulations. This study was approved by the Institutional Biosafety Committee of Chulalongkorn University (IBC No. 2131001) and Chulalongkorn University Animal Care and Use Protocol (CU-ACUP No. 2131004).

In addition, a longitudinal detection was performed for five cats that showed positive result in the initial DCH screening, namely PK-71, PK-74, PK-83, PK-91, and CU-38. The longitudinal study was done with a convenience-based sample collection of practitioners, and the duration of sample collection ranged from 13 to 111 days, depending on each cat's follow-up examination.

### Viral nucleic acid extraction and molecular screening for DCH

Approximately 200 μL of each specimen was subjected to a viral nucleic acid extraction procedure using the IndiSpin® Pathogen Kit (QIAGEN® GmbH, Germany) according to the manufacturer's protocol. The quality and quantity of the extracted nucleic acid were measured using a spectrophotometer (Nabi-UV/Vis Nano Spectrophotometer, Korea) at a 260/280 absorbance ratio. The extracted nucleic acid was then stored at −80°C until it was used further for viral molecular screening.

DCH screening was performed by a qPCR assay targeting the conserved overlapping region of P- and S-ORFs as previously described (11). Briefly, PCR master mix was prepared using the KAPA SYBR® FAST qPCR Master Mix kit (Kapa Biosystems, Sigma-Aldrich, South Africa) with the addition of a set of primers: DCH-qF (5′-CGTCATCATGGGCTTTAGGAA-3′) and DCH-qR (5′-TCCATATAAGCAAACACCATACAAT-3′). For the amplification of DCH DNA, the thermocycling conditions were set in the QIAGEN real-time PCR cycler Rotor-Gene Q (Qiagen, Germany) with *Taq* polymerase activation at 95°C for 3 min, followed by 35 cycles of denaturation at 95°C for 10 s and annealing at 60°C for 20 s. The amplification condition was then followed by the increasing the thermal cycler temperature from 70 to 95°C to acquire the melting curve from each amplicon. Samples that showed amplification curve above the threshold with single melting peak ranging from 80 to 85°C in accordance to the standard plasmids were considered as positive. The viral DNA copy number was obtained by comparing the acquired fluorescence signal from the samples with the standard plasmid containing the L-gene fragment of DCH (TOPO™ TA Cloning™ Kit with One Shot™ TOP10 Chemically Competent *E. coli*; Invitrogen, USA) designed using the available DCH sequences on GenBank (https://www.ncbi.nlm.nih.gov/genbank/) as previously described (6).

### Statistical analysis

All statistical analyses in this study were conducted using SAS statistical software version 9.4 (SAS Inst., Cary, NC, USA). Initially, the MEANS procedure was performed to analyze the descriptive statistical data including the mean, standard deviation (SD), minimum, and maximum DCH DNA level presented in each group of specimens. For cats with multiple DCH detections during the longitudinal sample collection and testing, only the single time point at which the level of DCH DNA in the blood was the highest was included for statistical analysis. Multiple analyses of variance were carried out using a general linear model procedure to compare the level of DCH DNA among groups of specimens.

In addition, 14 DCH viremic cats were further classified based on the DCH DNA level present in the blood according to the correlation between HBV DNA level in the blood and HBV DNA positivity in other specimens as previously described (15). Only 14 of 25 DCH viremic cats could be included in this analysis due to the lack of other body fluid specimens available from the remaining 11 cases. In this study, we divided the tested cats into three different groups: (1) low viral load group, with a DCH DNA level <5 log copies (LC)/mL; (2) high viral load group, with a DCH DNA level between 5 and 7 LC/mL; and (3) very high viral load group, with a DCH DNA level > 7 LC/mL. The association between DCH DNA level in the blood and the detection in other body fluid specimens was subsequently analyzed using Fisher's exact test, and relative risk (RR) was estimated using the FREQ procedure. A *P*-value < 0.05 was considered statistically significant for all tests.

### Complete genome sequencing and phylogenetic analysis

Genomic characterization and analysis were conducted to compare the complete genome sequences obtained from blood to those obtained from other body fluid specimens. We also compared the complete genome sequences obtained from cats for which DCH DNA was detected only in single specimen to those obtained from cats for which DCH DNA was detected in multiple specimens. A total of nine complete genome sequences were retrieved from seven blood samples, one OS sample, and one RS sample. These cases were selected based on the highest number of viral loads present in the specimens and represented the cases for which DCH DNA was detected from only one route and those with DCH DNA detected in multiple routes. Conventional PCR with the addition of three different sets of primers was employed to retrieve the DCH complete genome as previously described (4, 6). Retrieved sequences were then aligned and compared with the reference DCH sequences obtained from GenBank, using the freely available Molecular Evolutionary Genetic Analysis (MEGA) 7.0 software (http://www.megasoftware.net/). All sequences were then subjected to the construction of a maximum likelihood phylogenetic tree using bootstrap analysis with 1,000 replications. The HKY+G+I model was implemented as the best model based on the lowest Bayesian information criterion number from the best-fit model algorithm in the MEGA 7.0 software. A set of primers for DCH complete genome amplification and the previously described DCH sequences used for constructing a phylogenetic tree are presented in Supplementary Tables S2, S3.

## Results

### DCH detection from various body fluid specimens

A total of 1,209 clinical specimens were collected from 921 different cats. In detail, the clinical samples consisted of blood specimens collected from 532 cats, OS from 394 cats, RS from 182 cats, NS from 88 cats, AS from 8 cats, and urine from 5 cats. A total of 30/921 (3.26%) cats showed a positive result for DCH DNA detection by qPCR from at least one body fluid specimen. Within these 30 DCH-positive cats, 20 cats showed a positive result only in the blood (in 11/20 cats, only blood specimens were available; and in the other 9/20 cats, various body fluid specimens additional to the blood were available, but showed negative result). In addition to these 20 cases that showed positive only in the blood, four cats revealed positive detection only in the OS (blood specimens were not available in these cases), and six cats showed a positive result in more than one body fluid specimen. Of the six cats with DCH detection in more than one specimen, two cats (Case Nos. PK-95 and PK-98) revealed a positive result in the blood and OS; two other cats (Case Nos. PK-71 and PK-83) showed a positive result in the blood, OS, and RS; one cat (Case No. PK-91) revealed a positive result in the blood, OS, RS, and AS; and one cat (Case No. S-191) showed a positive result in the OS and NS (blood specimen was not available).

As for the clinical specimen level, the qPCR for DCH detection employed in this study revealed a positive result in 40/1,209 (3.31%) of the collected samples. Specifically, DCH DNA was detected in 25/532 (4.70%), 10/394 (2.54%), 3/182 (1.65%), 1/88 (1.14%), and 1/8 (12.5%) blood, OS, RS, NS, and AS specimens, respectively. No DCH-positive result was found in the urine specimens. The overall result of DCH detection in cats and the details of sample availability from every DCH-positive cat are presented in Table 1.

**Table 1:** DCH detection in various body fluid specimens with the details of sample's availability.

**Case no**	**DCH DNA copies number (log copies/mL) in clinical samples**					
	**Blood**	**OS**	**NS**	**AS**	**RS**	**Urine**
PT-57^a^	6.4	-	n/a	-	-	n/a
PK-71^a,b^	9.1	5.5	-	-	6.2	n/a
PK-74^a^	6.0	-	n/a	-	-	n/a
CU-37^a^	6.6	-	n/a	-	-	n/a
CU-38^a^	5.7	-	n/a	-	-	n/a
PK-83^a,b^	9.9	6.4	n/a	-	6.9	-
PK-91^a,b^	9.4	6.4	n/a	6.8	6.5	n/a
KB-18^a,b^	9.0	-	n/a	n/a	-	n/a
KB-19^a^	6.6	-	n/a	n/a	-	n/a
PK-92^a^	6.2	-	-	-	-	-
PK-93^a^	6.2	-	n/a	n/a	n/a	n/a
PK-94^a,b^	7.4	-	n/a	n/a	n/a	n/a
PK-95^a,b^	9.1	4.7	n/a	n/a	n/a	n/a
PK-98^a,b^	9.0	4.2	n/a	n/a	n/a	n/a
PB-45	5.8	n/a	n/a	n/a	n/a	n/a
PB-46	6.6	n/a	n/a	n/a	n/a	n/a
PB-71	6.0	n/a	n/a	n/a	n/a	n/a
PB-79^b^	9.3	n/a	n/a	n/a	n/a	n/a
PB-84	5.2	n/a	n/a	n/a	n/a	n/a
PB-87^b^	9.0	n/a	n/a	n/a	n/a	n/a
PB-88	3.6	n/a	n/a	n/a	n/a	n/a
PB-99^b^	9.6	n/a	n/a	n/a	n/a	n/a
SC-54^b^	7.4	n/a	n/a	n/a	n/a	n/a
SCKU-19^b^	8.6	n/a	n/a	n/a	n/a	n/a
SCKU-40^b^	8.2	n/a	n/a	n/a	n/a	n/a
S6-47	n/a	9.9	n/a	n/a	n/a	n/a
S6-58	n/a	9.1	n/a	n/a	n/a	n/a
PK-40	n/a	5.8	n/a	n/a	n/a	n/a
PK-41	n/a	6.1	n/a	n/a	n/a	n/a
S-191	n/a	8.4	9.2	n/a	n/a	n/a
**Total** ^**c**^	**25**	**10**	**1**	**1**	**3**	**0**

OS, oral swab; NS, nasal swab; AS, auricular swab; RS, rectal swab; n/a, not available; -, negative result.

a Cases where specimens additional to the blood were available.

b Cases where DCH DNA level in the blood were >7 log copies/mL.

c Total of positive cases in each specimen.

### DCH DNA quantification

**Figure 1:**
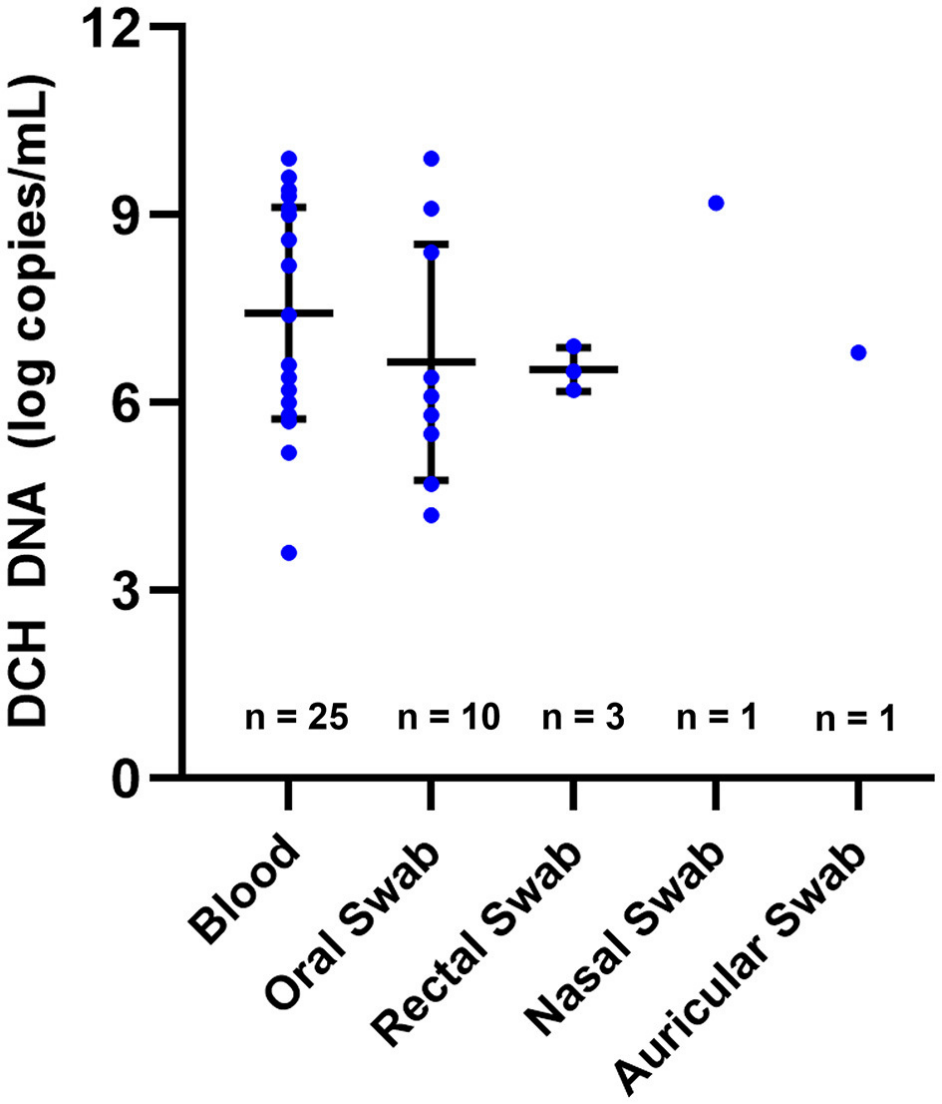
DCH DNA levels detected in various specimens. The level of DCH DNA detected in blood, oral swab, rectal swab, nasal swab, and auricular swab showed no significant difference. In this figure, the means and SDs are indicated by horizontal and vertical bars, respectively.

The level of DCH DNA detected in the blood ranged from 3.6 to 9.9 LC/mL, with a mean (±SD) of 7.4 ± 1.7 LC/mL. In the OS and RS specimens, DCH DNA was detected with copy numbers ranging from 4.2 to 9.9 LC/mL and 6.2 to 6.9 LC/mL, respectively, whereas the means (±SD) were 6.7 ± 1.9 LC/mL and 6.5 ± 0.3 LC/mL. As for the AS and NS specimens, a positive result was found in only one sample of each specimen, with a DNA level of 6.8 and 9.2 LC/mL, respectively (Figure 1). The multiple analyses of variance employed in this study revealed that there was no significant difference in the DCH DNA level between the blood, OS, RS, NS, and AS specimens.

Within the 25 DCH viremic cats, various body fluid specimens, in addition to the blood samples, were available only in 14 cats that were grouped evenly into the high viral load (7/14; 50%) and very high viral load (7/14; 50%) groups. Strikingly, a level of DCH DNA in the blood higher than 7 LC/mL (i.e., very high viral load group) was significantly correlated with DCH detection in the OS and RS specimens (*P* = 0.02; RR = 3.5 and *P* = 0.04; RR = 4, respectively), whereas there was no significant correlation between the level of DCH DNA in the blood with the presence of DCH DNA in the AS specimens (Table 2). NS specimen was excluded from the statistical analysis due to the lack of sample availability.

**Table 2:** The associations between DCH DNA level in blood with the detection in other specimens.

**DNA level in blood**	**DCH DNA detection in other specimens**			
	**OS** ^a^	**RS** ^b^	**AS**	**NS**
**High viral load group (5–7 log copies/mL)**				
PT-57	–	–	–	n/a
PK-74	–	–	–	n/a
CU-37	–	–	–	n/a
CU-38	–	–	–	n/a
KB-19	–	–	n/a	n/a
PK-92	–	n/a	n/a	n/a
PK-93	–	n/a	n/a	n/a
**Very high viral load group (**>**7 log copies/mL)**				
PK-71	+	+	–	–
PK-83	+	+	–	n/a
PK-91	+	+	+	n/a
KB-18	–	–	n/a	n/a
PK-94	–	n/a	n/a	n/a
PK-95	+	n/a	n/a	n/a
PK-98	+	n/a	n/a	n/a

OS, oral swab; NS, nasal swab; AS, auricular swab; RS, rectal swab; n/a, not available; +, positive result; –, negative result.

a Statistically significant (*P* = 0.02, RR = 3.5).

b Statistically significant (*P* = 0.04, RR = 4).

### Longitudinal detection of DCH

A total of 4/5 cats included in the longitudinal DCH detection study (PK-71, PK-74, PK-83, and PK-91) showed viremia throughout the collection period (13–111 days), whereas one cat (CU-38) showed DCH viremia only on the first sampling (day 0) and was negative on the next sampling (day 90). The DCH DNA level detected in the blood from all five cats during the longitudinal study ranged from 4.2 to 9.9 LC/mL. Broader detection of DCH in the specimens other than blood was observed from 3/5 cats (PK-71, PK-83, and PK-91), whereas no broader detection was observed from cats PK-74 and CU-38.

Furthermore, cat PK-71 showed DCH positivity in the OS on day 0 with a viral copy number of 4.8 LC/mL and was negative on the next sampling (day 23). On day 57, however, recurrent DCH positivity in the OS was observed, with a DCH DNA level of 5.5 LC/mL, and on day 111, the OS specimen tested negative. Other than the OS specimen, DCH DNA was also detected in the RS on day 57, with a viral copy number of 6.2 LC/mL. DCH detection was negative in the AS at three sample collection times (day 23, day 57, and day 111), and there were no NS and urine specimens available from cat PK-71.

Cat PK-83 was screened twice, and only blood and OS specimens were available at the first screening (day 0). Other than blood, DCH positivity was observed in the OS specimens throughout the sampling collection period. The viral copy numbers detected in the OS specimens were 6.6 and 6.4 LC/mL on day 0 and day 13, respectively. DCH DNA was also detected in the RS specimen on day 13 at a level of 6.5 LC/mL, and no detection was found in the AS and urine specimens. An NS specimen was not available during the longitudinal sample collection of cat PK-83.

Cat PK-91 was screened three times, on day 0, day 7, and day 55. DCH DNA was not detected in body fluid specimens other than blood on day 0 and day 7. In contrast, all available specimens, including blood, AS, OS, and RS, showed DCH positivity on day 55. The viral copy numbers detected in the AS, OS, and RS specimens on day 55 were 6.8, 6.4, and 6.5 LC/mL, respectively. No NS specimen was available from cat PK-91 throughout the sample collection period. The overall results of longitudinal DCH detection in this study are presented in Table 3.

**Table 3:** Longitudinal detection of DCH DNA from five different cases.

	**DCH DNA copies number (log copies/mL)**					
	**Clinical specimens**					
	**Blood**	**OS**	**NS**	**AS**	**RS**	**Urine**
**PK-71**						
05/03/2022 (Day 0)	8.2	4.8	n/a	n/a	n/a	n/a
15/03/2022 (Day 10)	8.2	n/a	n/a	n/a	n/a	n/a
28/03/2022 (Day 23)	8.3	-	n/a	-	-	n/a
01/05/2022 (Day 57)	9.1	5.5	n/a	-	6.2	n/a
24/06/2022 (Day 111)	8.1	-	n/a	-	-	n/a
**PK-74**						
05/03/2022 (Day 0)	4.2	-	n/a	n/a	n/a	n/a
15/03/2022 (Day 10)	6.0	n/a	n/a	n/a	n/a	n/a
24/03/2022 (Day 19)	5.7	-	n/a	-	-	n/a
**PK-83**						
25/04/2022 (Day 0)	9.7	6.6	n/a	n/a	n/a	n/a
08/05/2022 (Day 13)	9.9	6.4	n/a	-	6.5	-
**PK-91**						
04/05/2022 (Day 0)	8.3	-	n/a	n/a	n/a	n/a
11/05/2022 (Day 7)	9.0	-	n/a	-	-	n/a
28/06/2022 (Day 55)	9.4	6.4	n/a	6.8	6.5	n/a
**CU-38**						
13/03/2022 (Day 0)	5.7	-	n/a	-	-	n/a
11/06/2022 (Day 90)	-	-	n/a	-	-	n/a

OS, oral swab; NS, nasal swab; AS, auricular swab; RS, rectal swab; n/a, not available; -, negative result.

### Complete genome and phylogenetic analysis

Nine 3,184-bp complete genome sequences were successfully characterized from seven blood samples, designated as DCH/KB18-B/THA/2022, DCH/PK98-B/THA/2022, DCH/PK83-B/THA/2022, DCH/PK71-B/THA/2022, DCH/PK74-B/THA/2022, DCH/PK95-B/THA/2022, and DCH/PK91-B/THA/2022; one OS specimen, designated as DCH/S647-OS/THA/2016; and one RS specimen, designated as DCH/PK83-RS/THA/2022 (GenBank Accession Nos. OQ362106—OQ362114). All complete DCH sequences retrieved in this study showed a high percentage of nucleotide identity (96–100%) among themselves, and no distinctive sequence was observed (data not shown).

**Figure 2:**
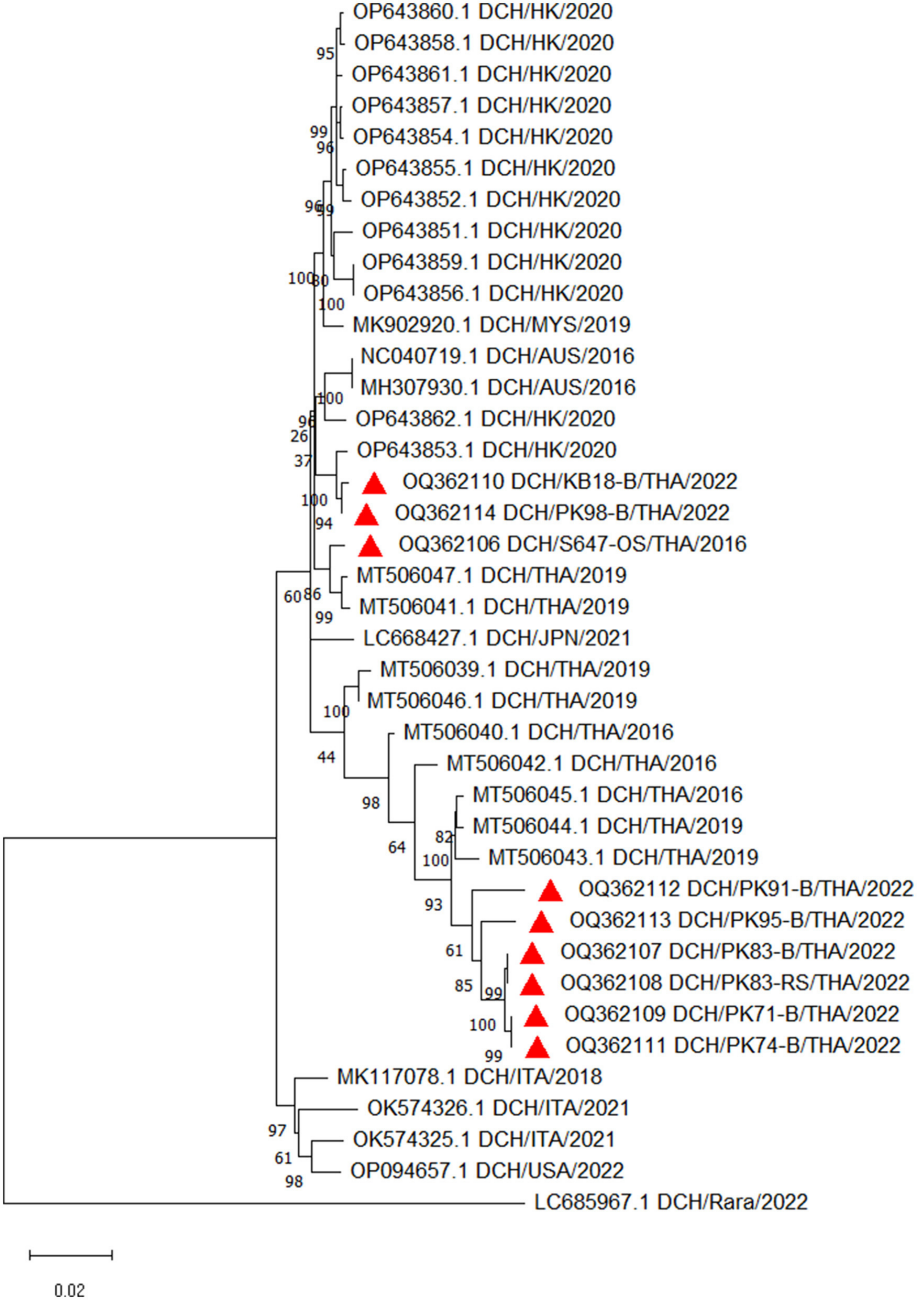
Phylogenetic tree constructed from the complete nucleotide sequences of DCH retrieved in this study. The maximum likelihood phylogenetic tree was constructed with the addition of available DCH sequences in GenBank and demonstrated that nine sequences obtained in this study (labeled with 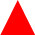) were grouped into two different lineages.

The constructed phylogenetic tree revealed that nine sequences from this study were grouped into two different lineages (Figure 2). Six sequences (OQ362107, OQ362108, OQ362109, OQ362111, OQ362113, and OQ362112) shared the same lineage with previous DCH strains from Thailand (Accession Nos. MT506042.1, MT506043.1, MT506044.1, and MT506045.1). In contrast, three other sequences (OQ362106, OQ362110, and OQ362114) were grouped into different lineages together with some DCH strains from Thailand (Accession Nos. MT506047.1 and MT506041.1) and DCH strains from Malaysia, Australia, Japan, Italy, and Hong Kong (Accession Nos. MK902920.1, MH307930.1, LC668427.1, OK574326.1, and OP643851.1—OP643862.1, respectively). In addition, the phylogram demonstrated that the complete nucleotide sequences obtained from cases where DCH DNA was detected only in the blood (Case Nos. KB-18 and PK-74; Accession Nos. OQ362110 and OQ362111) were not distinct from those cases that expressed DCH DNA in the blood and other specimens (Case Nos. PK-71, PK-83, PK-91, PK-95, and PK-98; Accession Nos. OQ362109, OQ362107, OQ362112, OQ362113, and OQ362114).

## Discussion

Hepadnaviruses have been long described to be associated with chronic hepatic diseases in some species, including humans and woodchucks (17, 18). In 2018, a novel hepadnavirus was first discovered in cats, tentatively named DCH (4). To date, several studies have been conducted to investigate the pathogenicity potential of DCH in feline species, and an association between DCH infection and liver disturbances in cats has been suggested (5, 6, 9, 11). The potential role of DCH in domestic cats, therefore, cannot be neglected.

In humans, horizontal transmission plays a contributive role in the increasing number of HBV infections, and concern about HBV transmissibility from various body fluid specimens other than blood has been raised in the last decade (12, 19). This concern was especially highlighted in settings where close contact with chronically infected patients that showed no apparent clinical signs was inevitable, such in children's daycares or preliminary schools (12, 13). Correspondingly, the detection of HBV DNA in various body fluid specimens other than blood, including cerumen, sweat, nasopharyngeal swab, urine, saliva, and tears, has been reported in numerous studies (3, 12, 14, 15, 20, 21). In contrast to the broad evidence demonstrating the detection of HBV DNA in various body fluid specimens from infected patients, to date, there has been no report on the detection of DCH DNA in body fluid specimens other than blood in cats, although such an investigation was attempted in an earlier study (16). In this study, therefore, we investigated the detection of DCH DNA in blood and other body fluid specimens of cats. Notably, our result showed that DCH DNA could be detected in various body fluid specimens, including the blood, AS, NS, OS, and RS, whereas no positive detection was observed in urine. The low number of urine samples availability might explain the absence of DCH positivity from urine specimen in this study, however, the detection of other hepadnavirus from urine specimens has been documented in several studies (22, 23).

The prevalence of DCH DNA detected in clinical samples in this study ranged from 1.14 to 4.70%, with the lowest prevalence observed in the NS specimens and highest prevalence obtained from blood. In HBV, broader detection of viral DNA in various body fluid specimens has been evidenced in several studies, with saliva and semen first experimentally demonstrated to be infectious (20). Later, *in vivo* experiment showed that the tears of chronically infected patients are also considered to be highly infectious (12). In regard to the risk of horizontal transmission of HBV from various routes other than blood, with the addition of the increasing trend of multi-cat settings where fighting and close contact between cats are inevitable, the detection of DCH DNA in various specimens in this study is enthralling. To what extent the detectable DCH DNA from various routes plays a role in infectivity and horizontal transmission in cat populations, however, requires further experimental studies.

The infectious potential of hepadnaviruses is also directly correlated with expressed viral load in the specimens (12). In this study, the mean DCH viral load detected in the blood, AS, NS, OS, and RS was >6 LC/mL, whereas in HBV, it has been described that body fluids containing HBV DNA > 5 LC/mL have the potential to be transmission vehicles, especially in highly endemic areas (12, 14, 15). The infectious risk of body fluids such as saliva and nasopharyngeal swab specimens containing high titers of HVB DNA has also been highlighted in multi-individual settings, such as those in childcare vicinities where horizontal transmission among children has been concerning (24). Although the result in which DCH DNA was detected in high titers from various body fluid specimens is intriguing, the clinical implications is yet to be explained because the DCH prevalence in this study is also lower compared with that observed in previous studies in Thailand (6).

In this study, it must be noted that although the sample collection procedures were performed with extra caution to prevent any trauma, the presence of blood from previously existing trauma and/or microtrauma could not be ruled out. However, during longitudinal sample testing, the OS specimens collected from cat number PK-71 revealed recurrent DCH DNA positivity (on the fourth testing) together with the increased DNA level in the blood and was then negative again on the next testing with a decreased DNA level in the blood (Table 3). Therefore, the presence of DCH DNA in body fluid specimens other than blood in this case might indicate the hematogenous spreading of DCH DNA. This result agrees with the evidence in humans showing that HBV viral DNA can be independently present in cerumen specimens without any blood contamination, as they ruled out the presence of blood in the collected specimens using a specific Meyer test (21).

It is also worth noting that of 14 cats in which various specimens in addition to the blood were available, the detection of DCH DNA in the OS, RS, NS, and AS was only observed in the group containing more than 7 LC/mL in the blood, whereas no detection was observed in the group where DCH DNA in the blood ranged between 5 and 7 LC/mL or less. A previous study investigating the presence of DCH DNA in oral, conjunctival, preputial, and rectal swabs also revealed negative results in all specimens collected from cats with DCH DNA levels in the blood ranging from 5.2 to 6.3 LC/mL (16). Correspondingly, there were positive correlations between the level of DCH DNA in the blood and the detection in OS and RS specimens. Statistically, this study revealed that DCH viremia cats with DCH DNA levels in the blood higher than 7 LC/mL are 3.5 and 4 times more likely to express DCH DNA in OS and RS specimens, respectively. The result in this study raised supporting evidence that mirrors the characteristic of HBV, where a correlation between the presence of high levels of HBV DNA in the serum with the presence of HBV DNA in other body fluids such as cerumen, saliva, nasopharyngeal swab, tears, and sweat has been documented in chronically infected humans (12, 21, 24–26). The increasing level of HBV DNA in the blood of HBV chronically infected patients is commonly seen during the HBV reactivation period due to various underlying causes that promotes immunosuppressive conditions, such as prior human immunodeficiency virus infection, chronic diseases, therapeutic intervention and/or chemotherapy, and inadequate host immune response (27). In this study, the direct correlation between the clinical presentations and immunosuppression status of the cats with DCH DNA levels in the serum could not be drawn due to the limited history at the time of sample collection and warrants further investigation.

In contrast to the OS and RS, there was no positive correlation between the viral load in the blood with the detection of DCH DNA in the AS specimens. This result might represent the real number of detections in the specimens, which was very low, and/or could be due to the limited number of samples. As for the association between DCH viral load in the blood with the presence of DCH DNA in NS specimen, no correlation could be determined due to the lack of sample availability. Because this is the only study describing DCH DNA detection in various body fluid specimens other than blood in cats, broader epidemiological studies are needed to further elucidate the correlation between the presence of high viral titers in the blood and the expression of DCH DNA in other body fluids in cats.

In addition, this study revealed that four out of five cats included in the longitudinal study remained positive for DCH DNA detection in the blood for the whole timeline of sample collection, ranging from 1 to 4 months. Longitudinal detection of DCH DNA in the blood was also documented in a previous study by Capozza et al., who detected DCH DNA in the blood of a DCH-positive cat for as long as 11 months (16). HBV DNA detection in serum implies some clinical importance in humans, including the diagnosis of reactivity in chronic infection, as well as an independent risk predictor of HCC development when coupled with the observation of alanine aminotransferase and aspartate aminotransferase (28, 29).

Finally, the phylogenetic analysis in this study revealed that all complete genome sequences obtained from blood, OS, and RS specimens showed a high percentage of identity among themselves. Moreover, there was no significant genomic distance between the complete genome sequences obtained from cases where DCH DNA was present only in the blood, compared with cases with DCH detection in blood and other routes. This result suggests that genomic diversity would not likely play a role in the DCH DNA positivity in other body fluid specimens. The result in this study supports a previous result where HBV DNA was detected in saliva and nasopharyngeal swabs of children with chronic infection, but an HBV genotypic difference was not presented in these patients (24). Although different HBV genotypes promote different disease progression, they are unlikely to affect the expression of viral DNA in the body fluid specimens (30).

In conclusion, this study revealed the detection of DCH DNA in various body fluid specimens of cats, including the blood, AS, NS, OS, and RS. The risk of cats expressing DCH DNA in the OS and RS was significantly higher when the DCH DNA level presented in the blood was >7 LC/mL. The relatively high level of DCH DNA in various specimens in addition to the blood might represent true expression of the viral DNA or the presence of blood due to trauma during or prior to sample collection. However, in accordance with the direct correlation between the DNA level in the specimens and transmissibility potential, the infectious risk from various specimens, if any, cannot be neglected. The detection of DCH DNA in broader routes was not associated with any genomic diversity. To what extent the expression of DCH DNA in various body fluid specimens of cats plays role in horizontal transmission in cat populations warrants further investigation.

## Data availability statement

The datasets presented in this study can be found in online repositories. The names of the repository/repositories and accession number(s) can be found below: https://www.ncbi.nlm.nih.gov/; OQ362106—OQ362114.

## Ethics statement

The animal studies were approved by Institutional Biosafety Committee of Chulalongkorn University (IBC No. 2131001) and Chulalongkorn University Animal Care and Use Protocol (CU-ACUP No. 2131004). The studies were conducted in accordance with the local legislation and institutional requirements. Written informed consent was obtained from the owners for the participation of their animals in this study.

## Author contributions

CP and ST designed the research and finalized the manuscript. SWW conducted the experiments, analyzed the result, and wrote the first draft of manuscript. PT performed statistical analysis. All authors read and approved the final manuscript.

## References

[1] Tsukuda S Watashi K. Hepatitis B virus biology and life cycle. Antiviral Res. (2020) 182:104925. 10.1016/j.antiviral.2020.104925 32866519

[2] Karayiannis P. Hepatitis B virus: virology, molecular biology, life cycle and intrahepatic spread. Hepatol Int. (2017) 11:500–8. 10.1007/s12072-017-9829-7 29098564

[3] Meo SA Assad AA Sanie FM Baksh ND Al-Qahtani A Shaikh ZA. Transmission of hepatitis-B virus through salivary blood group antigens in saliva. J Coll Physicians Surg Pak. (2010) 20:444–8. 20642943

[4] Aghazadeh M Shi M Barrs VR McLuckie AJ Lindsay SA Jameson B. A novel hepadnavirus identified in an immunocompromised domestic cat in Australia. Viruses. (2018) 10:269. 10.3390/v10050269 29772771 PMC5977262

[5] Pesavento PA Jackson K Hampson TSTTB Munday JS Barrs VR Beatty JA. A novel hepadnavirus is associated with chronic hepatitis and hepatocellular carcinoma in cats. Viruses. (2019) 11:969. 10.3390/v11100969 31640283 PMC6832243

[6] Piewbang C Wardhani SW Chaiyasak S Yostawonkul J Chai-in P Boonrungsiman S. Insights into the genetic diversity, recombination, and systemic infections with evidence of intracellular maturation of hepadnavirus in cats. PLoS ONE. (2020) 15:e0241212. 10.1371/journal.pone.0241212 33095800 PMC7584178

[7] Anpuanandam K Selvarajah GT Choy MMK Ng SW Kumar K Ali RM. Molecular detection and characterisation of Domestic Cat Hepadnavirus (DCH) from blood and liver tissues of cats in Malaysia. BMC Vet Res. (2021) 17:9. 10.1186/s12917-020-02700-0 33407487 PMC7788742

[8] Jeanes EC Wegg ML Mitchell JA Priestnall SL Fleming L Dawson C. Comparison of the prevalence of Domestic Cat Hepadnavirus in a population of cats with uveitis and in a healthy blood donor cat population in the United Kingdom. Vet, Opthalmol. (2022) 25:165–72. 10.1111/vop.12956 34806802

[9] Piewbang C Dankaona W Poonsin P Yostawonkul J Lacharoje S Sirivisoot S. Domestic cat hepadnavirus associated with hepatopathy in cats: a retrospective study. J Vet Intern Med. (2022) 36:1648–59. 10.1111/jvim.16525 36054642 PMC9511090

[10] Takahashi K Kaneko Y Shibanai A Yamamoto S Katagiri A Osuga T. Identification of domestic cat hepadnavirus from a cat blood sample in Japan. J Vet Med Sci. (2022) 84:648–52. 10.1292/jvms.22-0010 35321970 PMC9177394

[11] Lanave G Capozza P Diakoudi G Catella C Catucci L Ghergo P. Identification of hepadnavirus in the sera of cats. Sci Rep. (2019) 9:10668. 10.1038/s41598-019-47175-8 31337847 PMC6650429

[12] Komatsu H Inui A Sogo T Tateno A Shimokawa R Fujisawa T. Tears from children with chronic hepatitis B virus (HBV) infection are infectious vehicles of HBV transmission: experimental transmission of HBV by tears, using mice with chimeric human livers. J Infect Dis. (2012) 206:478–85. 10.1093/infdis/jis293 22508939

[13] Heiberg IL Hoegh M Ladelund S Niesters HGM Hogh B. Hepatitis B virus DNA in saliva from children with chronic hepatitis B infection: implications for saliva as a potential mode of horizontal transmission. J Pediatr Infect Dis. (2010) 29:465–7. 10.1097/INF.0b013e3181d8e009 20335824

[14] Gholami-Parizad E Taherikalani M Mozaffar-Sabet NA Asmar M Gholami-Parizad S Khosravi A. Cerumen as a potential risk for transmission of Hepatitis B virus. Acta Microbiol Immunol Hung. (2011) 58:105–12. 10.1556/amicr.58.2011.2.3 21715280

[15] Gholami Parizad E Gholami Parizad E Khosravi A Amraei M Valizadeh A Davoudian A. Comparing HBV viral load in serum, cerumen, and saliva and correlation with HBeAg serum status in patients with chronic hepatitis B infection. Hepat Mon. (2016) 16:e30385. 10.5812/hepatmon.30385 27313632 PMC4908613

[16] Capozza P Lanave G Diakoudi G Stasi F Ghergo P Ricci D. A longitudinal observational study in two cats naturally-infected with hepadnavirus. Vet Microbiol. (2021) 254:108999. 10.1016/j.vetmic.2021.108999 33524809

[17] Ryu W-S. Chapter 18 - Hepadnaviruses. In: Ryu W-S, editor. Molecular Virology of Human Pathogenic Viruses. Boston, MA: Academic Press (2017). p. 247–60.

[18] Hu K-Q. Hepadnaviruses: Virological and Clinical Features. Reference Module in Biomedical Sciences. Amsterdam: Elsevier (2019). p. 1–9.

[19] Komatsu H Inui A Sogo T Hiejima E Kudo N Fujisawa T. Source of transmission in children with chronic hepatitis B infection after the implementation of a strategy for prevention in those at high risk. Hepatol Res. (2009) 39:569–76. 10.1111/j.1872-034X.2009.00496.x 19260997

[20] Scott RM Snitbhan R Bancroft WH Alter HJ Tingpalapong M. Experimental transmission of hepatitis B virus by semen and saliva. J Infect Dis. (1980) 142:67–71. 10.1093/infdis/142.1.67 7400629

[21] Eftekharian A Moghaddasi H Gachkar L Amlashi SSA. Detection of hepatitis B virus in the cerumen of patients with chronic hepatitis B infection. J Laryngol Otol. (2013) 127:1065–6. 10.1017/S0022215113002314 24131958

[22] Jain S Su Y-H Su Y-P McCloud S Xue R Lee T-J. Characterization of the hepatitis B virus DNA detected in urine of chronic hepatitis B patients. BMC Gastroenterol. (2018) 18:40. 10.1186/s12876-018-0767-1 29548283 PMC5857095

[23] Lin SY Su YP Trauger ER Song BP Thompson EGC Hoffman MC. Detection of hepatitis B virus-host junction sequences in urine of infected patients. Hepatol Commun. (2021) 5:1649–59. 10.1002/hep4.1783 34558837 PMC8485884

[24] Kidd-Ljunggren K Holmberg A Bläckberg J Lindqvist B. High levels of hepatitis B virus DNA in body fluids from chronic carriers. J Hosp Infect. (2006) 64:352–7. 10.1016/j.jhin.2006.06.029 17046105

[25] Kalcioglu MT Durmaz R Ozturan O Bayindir Y Direkel S. Does cerumen have a risk for transmission of hepatitis B? Laryngoscope. (2004) 114:577–80. 10.1097/00005537-200403000-00035 15091238

[26] Goh EK Son BH Kong SK Chon KM Cho KS. Analysis of hepatitis B virus in the cerumen and otorrhea of chronic HBV-infected patients: is there a hepatitis B virus infectivity? Otol Neurotol. (2008) 29:929–32. 10.1097/MAO.0b013e31817fdfc3 18665006

[27] Chang Y Jeong SW Jang JY. Hepatitis B virus reactivation associated with therapeutic interventions. Front Med. (2022) 8:770124. 10.3389/fmed.2021.770124 35096867 PMC8795508

[28] Chen CF Lee WC Yang HI Chang HC Jen CL Iloeje UH. Changes in serum levels of HBV DNA and alanine aminotransferase determine risk for hepatocellular carcinoma. Gastroenterology. (2011) 141:1240–8.e2. 10.1053/j.gastro.2011.06.036 21703214

[29] Xu X Jiang J Song C Yu C Zhu L Qian J. Association of dynamic changes in serum levels of HBV DNA and risk of hepatocellular carcinoma. Curr Med. (2022) 1:5. 10.1007/s44194-022-00008-9

[30] Sunbul M. Hepatitis B virus genotypes: global distribution and clinical importance. World J Gastroenterol. (2014) 20:5427–34. 10.3748/wjg.v20.i18.5427 24833873 PMC4017058

