# Supplementary material for: Quantification of domestic cat hepadnavirus DNA in various body fluid specimens of cats: the potential viral shedding routes
Source: Front Vet Sci. 2023 Sep 4;10:1248445. doi: 10.3389/fvets.2023.1248445 (PMC10507184; doi:10.3389/fvets.2023.1248445)
Supplement: Supplementary file 1 [file Table_1.DOCX]

Supplementary Material

Quantification of domestic cat hepadnavirus (DCH) DNA in various body fluid specimens of cats: the potential viral shedding routes

**Sabrina Wahyu Wardhani^1,2^, Padet Tummaruk^3^, Chutchai Piewbang^2,4,*^, Somporn Techangamsuwan^2,4,*^**

^1^The International Graduate Program of Veterinary Science and Technology (VST), Faculty of Veterinary Science, Chulalongkorn University, Bangkok 10330, Thailand

^2^Animal Virome and Diagnostic Development Research Unit, Faculty of Veterinary Science, Chulalongkorn University, Bangkok 10330, Thailand

^3^Department of Obstetrics, Gynaecology, and Reproduction, Faculty of Veterinary Science, Chulalongkorn University, Bangkok, 10330, Thailand

^4^Department of Pathology, Faculty of Veterinary Science, Chulalongkorn University, Bangkok, 10330, Thailand

* These authors are correspondences and contributed equally to this work

***Correspondences**: Dr. Chutchai Piewbang, Department of Pathology, Faculty of Veterinary Science, Chulalongkorn University, Bangkok 10330, Thailand

Associate Professor Dr. Somporn Techangamsuwan, Department of Pathology, Faculty of Veterinary Science, Chulalongkorn University, Bangkok 10330, Thailand

E-mail: alkaline_eart@hotmail.com; somporn.t@chula.ac.th

# Supplementary table S2. Primer sets used to obtain the complete genome of DCH

| **Primer name** | **Primer sequences 5’-3’** | **Target size (bp)** | **Tm (°C)** | **Reference** |
| --- | --- | --- | --- | --- |
| DCH-P1F | TTGGCACCTGGATTCGCA | 1699 | 57 | Alghazadet, et al., 2018 |
| DCH-P1R1 | AGATGTTCCACACTCTTAGCC |  |  |  |
| DCH-P2F1 | ATCCCGTCATCATGGGCTT | 830 | 55 | Alghazadet, et al., 2018; Piewbang, et al., 2020 |
| DCH-P2R1 | GGACGTAGACGAAGGACACGT |  |  |  |
| DCH-P2F | CCATCGATTTACACACTTCCCA | 997 | 55 | Alghazadet, et al., 2018; Piewbang, et al., 2020 |

# Supplementary table S3. DCH sequences from this study and reference sequences used to construct phylogenetic tree

| **Accession no.**  **(Sequence name in this study)** | **Origin** | **Year collected** |
| --- | --- | --- |
| OQ362106  (DCH/BKKS647-OS/THA/2016) | Thailand | 2016 |
| OQ362107  (DCH/PK83-B/THA/2022) | Thailand | 2022 |
| OQ362108  (DCH/KB83-RS/THA/2022) | Thailand | 2022 |
| OQ362109  (DCH/PK71-B/THA/2022) | Thailand | 2022 |
| OQ362110  (DCH/KB18-B/THA/2022) | Thailand | 2022 |
| OQ362111  (DCH/PK74-B/THA/2022) | Thailand | 2022 |
| OQ362112  (DCH/PK91-B/THA/2022) | Thailand | 2022 |
| OQ362113  (DCH/PK95-B/THA/2022) | Thailand | 2022 |
| OQ362114  (DCH/PK98-B/THA/2022) | Thailand | 2022 |
| NC040719.1 | Australia | 2016 |
| MH307930.1 | Australia | 2016 |
| MK902920.1 | Malaysia | 2019 |
| LC668427.1 | Japan | 2021 |
| LC685967.1 | Japan | 2022 |
| OP094657.1 | USA | 2022 |
| MK117078.1 | Italy | 2018 |
| OK574326. | Italy | 2021 |
| OK574325.1 | Italy | 2021 |
| MT506040.1 | Thailand | 2016 |
| MT506042.1 | Thailand | 2016 |
| MT506045.1 | Thailand | 2016 |
| MT506039.1 | Thailand | 2019 |
| MT506041.1 | Thailand | 2019 |
| MT506043.1 | Thailand | 2019 |
| MT506044.1 | Thailand | 2019 |
| MT506046.1 | Thailand | 2019 |
| MT506047.1 | Thailand | 2019 |
| OP643851.1 | Hongkong | 2020 |
| OP643852.1 | Hongkong | 2020 |
| OP643853.1 | Hongkong | 2020 |
| OP643854.1 | Hongkong | 2020 |
| OP643855.1 | Hongkong | 2020 |
| OP643856.1 | Hongkong | 2020 |
| OP643857.1 | Hongkong | 2020 |
| OP643858.1 | Hongkong | 2020 |
| OP643859.1 | Hongkong | 2020 |
